# Iterative reconstruction of industrial positron images with generative networks

**DOI:** 10.1371/journal.pone.0335912

**Published:** 2025-11-19

**Authors:** Mingwei Zhu, Min Zhao, Min Yao

**Affiliations:** 1 School of Mechanical Engineering, Nantong University, Nantong, Jiangsu, China; 2 College of Automation Engineering, Nanjing University of Aeronautics and Astronautics, Nanjing, Jiangsu, China; Dalian Maritime University, CHINA

## Abstract

Positron imaging has shown great potential in industrial non-destructive testing due to its high sensitivity and ability to reveal internal structures of complex components. However, reconstructing high-quality images from positron emission data remains challenging, particularly under limited sampling and ill-posed inverse problems, which are common in applications such as closed cavity detection. To address this, we propose an iterative reconstruction method for industrial positron images based on a generative adversarial network (PIIR-GAN). The method integrates a generative adversarial framework with a self-attention mechanism to exploit prior information and improve image quality under low-sample conditions. A key innovation is embedding the neural network model directly into the iterative reconstruction process, enabling end-to-end learning. Furthermore, a likelihood-based constraint is incorporated into the objective function to guide optimization. Experimental results on a GATE simulation dataset show significant improvements in both PSNR and SSIM compared with conventional methods, and real-world industrial defect detection further verifies the effectiveness of the approach.

## Introduction

Positron Emission Computed Tomography (PET) is functional imaging technology which is highly sensitive. Compared with other traditional industrial non-destructive testing method, such as X-ray and CT, the gamma photons produced in positron annihilation process have stronger penetrability and lower radiation. Therefore, it has a better application prospect in the detection of industrial airtight cavity with high precision.

The quality of PET image reconstruction largely depends on the number of response lines, which directly determines the amount of useful information. However, under current industrial conditions, available samples are limited, and the acquisition process often suffers from noise, sparse sampling, and environmental interference. Due to the ill-posed nature of the inverse problem, artifacts and noise frequently degrade the final image, reducing its utility in real-world industrial inspections.

Therefore, improving the PET imaging effect has become a current research focus for the application of PET technology in non-destructive testing of industrial defects. Existing research on PET image reconstruction mainly focuses on two aspects: one is processing the image in the post-reconstruction stage to enhance image quality [1–4]; the other is improving image quality by incorporating prior knowledge [5–8]. Methods of the latter type act directly on the reconstruction stage of positron images, better preserving the data obtained from sampling and thereby improving image quality. Although some studies on PET image reconstruction have made progress, most of these methods are applied to medical PET images. In industrial applications, however, due to constraints such as field environment, hardware limitations, and sampling efficiency, the performance of these methods in practical non-destructive testing remains unsatisfactory.

To address this issue, in the paper we discuss a reconstruction model for industrial positron images based on deep learning, building upon existing related studies. Specifically, we propose an approach to improve the quality of iterative reconstruction using Generative Adversarial Networks (GANs) [9] under low-sampling conditions in industrial settings. The method enables the acquisition of higher-quality defect detection images in complex industrial environments. Experimental results on both simulated and real images demonstrate that the proposed approach significantly improves image quality. Compared to existing deep learning-based PET reconstruction methods, which are primarily developed for medical imaging with relatively clean and abundant data, our approach is designed to handle the challenging conditions of industrial applications. Unlike typical post-processing or denoising methods, our model embeds prior knowledge directly into the iterative reconstruction process, preserving more sampling details and achieving higher accuracy under low-sample, high-noise conditions.

In this study, we discuss the mathematical model in the positron imaging process firstly. Then we use GAN to iteratively reconstruct the images. Finally, we conduct various experiments to determine the effects of adopting deep learning model in the reconstruction.

The main contributions of this paper are as follows.

- Get the prior input of the image reconstruction by training a small number of simulation data samples;
- Incorporated the deep neural network in the image iterative reconstruction stage to avoid the loss of sampling features.
- Optimize the neural network structure based on the combination of perceptual loss function and attention loss function.

The paper is organized as follows. The Related work section introduces the related work. The Method section describes the method we proposed in the paper. The Experiment section shows the experimental results and discusses the results in the discussion section. Finally, we provide conclusions in the last Section.

## Related work

In recent years, the application of deep learning in the field of computer vision has made a lot of progress. Compared with traditional methods, the deep neural network model has obvious performance improvement in image classification, image denoising, object detection, semantic segmentation and etc. Meanwhile, deep neural networks, which used to reconstruct images is also a research hotpot in the field of imaging. For example, deep convolutional networks were used to adjust the coefficients of wavelet transform and reduced the reconstruction noise of low-dose CT images [10]. On the basis of the above framework, a wavelet residual network denoising algorithm is proposed, which has good performance in preserving image texture details [11]. [12] presented a residual network model which combine auto-encoder and deconvolution networks to realize the structure fidelity and noise suppression of low-dose CT images. In PET imaging, [13] proposed a reconstruction method using a dynamic convolutional module that maps low-dose PET plus CT images into standard-dose PET. [14] presented an unsupervised CNN that uses cross-multiplication regularization on list-mode data to improve positron image reconstruction accuracy. [15] introduced LegoPET, a conditional diffusion model guided by hierarchical features for PET reconstruction from sinograms, outperforming prior methods in PSNR/SSIM. [16] proposed DREAM, which integrates random masks in both sinogram and latent spaces to better capture both local details and global structures.

The most of the common models on deep neural networks are constructed based on large-scale training data. However, when PET is used for industrial non-destructive testing, the sample data obtained under current conditional is less. Therefore, in order to get better reconstructed images, GANs are considered in the process of image reconstruction. It is a deep learning method based on probability and statistics theory to generate data samples. It does not require or only needs less labeled data to learn from the idea of game theory for efficient data generation. The model consists of generator and discriminator. The generator generates data and the discriminator judges the true or false, then the error of the discriminant result is passed to the network to improve the parameters. The above process is repeated continuously to optimize the performance of the model until it reached the Nash equilibrium stat, and the mathematical expression is as Eq (1).

$$\min_{G}\max_{D}V(G,D)=\min_{G}\max_{D}E_{x\sim P_{\text{data }}}[\log D(x)]+E_{z\sim P_{z}}[\log(1-D(G(Z)))]$$ (1)

Given the strong potential of GANs in generative modeling, many works have adapted GANs for image reconstruction tasks. For instance, [17] added a latent code in the random noise input to control the data generation, and added a mutual information regular term to indicate the degree of association. [18] combined the advantages of SRGAN (Super-Resolution Generative Adversarial Network) [19] and RaGAN (Relativistic Average Generative Adversarial Network) [20]. It used residual dense block units and a relative average discriminator to make the edges of the reconstructed images are sharper. [21] used the general reconstruction loss, gradient loss and additional adversarial loss to train full convolution network, and it successfully synthesized high quality real images. [22] proposed a distributed network, the first one synthesizes images, and the second used image translation framework to obtain higher resolution. [23] built a dual channel generative network to get more realistic global output based on conditional generative adversarial network. [24] proposed a controllable GAN based on the combination with generator of ResNet-like and discriminator of PatchGAN [25] and realized the faithful reconstruction of images. GAN and autoencoder were combined for image reconstruction and the model trained positive samples to build the images based on using local binary pattern for image local contrast to detect defects [26]. [27] proposed a self-supervised adaptive residual GAN (SS-AEGAN) that mitigates texture inconsistencies and enhances low-dose PET image quality through adaptive residual mapping and self-supervised pretraining. [28] proposed PCC-GAN, a point-based GAN that enhances PET reconstruction quality by capturing geometric and contextual relationships from low-dose data.

At the same time, to obtain better model training effects, more model regularization and generalization researches are also being further developed in the field of deep learning. [29] proposed a spectrum interference-based two-level data augmentation method in deep learning for automatic modulation classification. [30] optimized the specifically designed autoencoder (AE) by entropy-stochastic gradient descent. [31] improved he pruning method by reducing the network parameters and the calculation cost.

In summary, a number of studies have been conducted to overcome the challenging in image reconstruction, however, several aspects thereof have been not yet been satisfactorily resolved. In this study, we focus on addressing the following problem of images reconstruction under industrial positron imaging conditions. (i) the data range of the sample is small, (ii) data limitation under limited conditions, (iii) image quality is not very satisfactory. To solve these limitations, we decided to employ a GAN-based iterative reconstruction approach to obtain higher quality PET images, particularly in a more complex industrial environment.

## Method

### PET data model

The process of producing *γ* photons by positron annihilation can be abstracted into a Poisson distribution model and the mean value depends on the distribution of radionuclides. The goal of PET image reconstruction is to obtain the nuclide position distribution function in relative space. The measured data $y\in {\mathbb{R}}^{N\times 1}$ can be modeled as a collection of independent Poisson distribution and its mean $\overset{―}{y}\in {\mathbb{R}}^{N\times 1}$ is related to the image $x\in {\mathbb{R}}^{M\times 1}$ through statistical iteration. The model can be abstracted Eq (2).

$$\overset{―}{y}=Ax+s+r$$ (2)

Where $A\in {\mathbb{R}}^{M\times N}$ is the system matrix, in which *A*_*i*,*j*_ represents the photons originating from voxel *j* and detected by detector *i*. $s\in {\mathbb{R}}^{M\times 1}$ denotes the expectation of scattered events. $s\in {\mathbb{R}}^{M\times 1}$ denotes expectation of random coincidences. M is the number of lines of response (LOR) and *N* is the value of pixel in image space. The log-likelihood function can be written as Eq (3).

$$L(y|x)=\sum_{i=1}^{N}y_{i}\log{\bar{y}}_{i}-{\bar{y}}_{l}-\log y_{i}!$$ (3)

### Reconstruction model

The reconstructed image *x*, which mentioned above is considered to be represented by the model as Eq (4).

x=f(α) (4)

Where f:ℝ→ℝ denotes the neural network, and *α* is defined as the input to the adversarial network. By training the neural network with existing data, prior knowledge of industrial positron images is incorporated into the reconstruction framework.

And the maximum likelihood estimates of the images *x* is as Eq (5).

$$\hat{x}=\arg\max_{x\geq 0}L(y|x)$$ (5)

To improve reconstruction accuracy, we introduce an additional likelihood term to adjust the dimensionality of the random input *α* to match the feature scale of positron images. Then the maximum likelihood estimation of the image *x* can be calculated as Eq (6).

$$\hat{x}=\arg\max_{x\geq 0}\lambda L(y|A\alpha +s+r)+L(y|Ax+s+r)\;s.t.x=f(\alpha )$$ (6)

Here, the hyperparameter *λ* is used to balance the *α* and *x* to ensure the stability of the model and both of them are matched the sampled positron data.We constrain the network optimization with different values of *λ*, and after multiple training experiments, λ=0.5 was found to achieve stable results.

Then we use the Augmented Lagrange method to optimize the constraint problem above and the expression is as Eq (7).

$$L_{\rho }=\lambda L(y|A\alpha +s+r)+L(y|Ax+s+r)-\frac{\rho }{2}{\left\|X-f(\alpha )+\mu \right\|}^{2}+\frac{\rho }{2}{\left\|\mu \right\|}^{2}$$ (7)

And this expression can be solved by ADMM (Alternating direction method of multipliers) algorithm as Eq (8).

$$x^{n+1}=\arg\max_{x}L(y|Ax+s+r)-\frac{\rho }{2}{\left\|X-f(\alpha^{n})+\mu^{n}\right\|}^{2}\;\alpha^{n+1}=\arg\min_{\alpha }(y|A\alpha +s+r){\left\|f(\alpha )-(x^{n+1}+\mu^{n}\right\|}^{2}\;\mu^{n+1}=\mu^{n}+x^{n+1}-f(\alpha^{n+1})$$ (8)

where *μ* denotes the Lagrange multiplier introduced in the augmented Lagrangian formulation, which is iteratively updated in the ADMM optimization process to enforce the constraint x=f(α).

The reconstruction framework can include the constraint through pre-trained adversarial network model using existing images.We adopt a GAN-based structure as the backbone network, considering its ability to learn low-dimensional manifolds of high-count samples and achieve better performance than conventional convolutional networks under limited sampling conditions.

**Fig 1:**
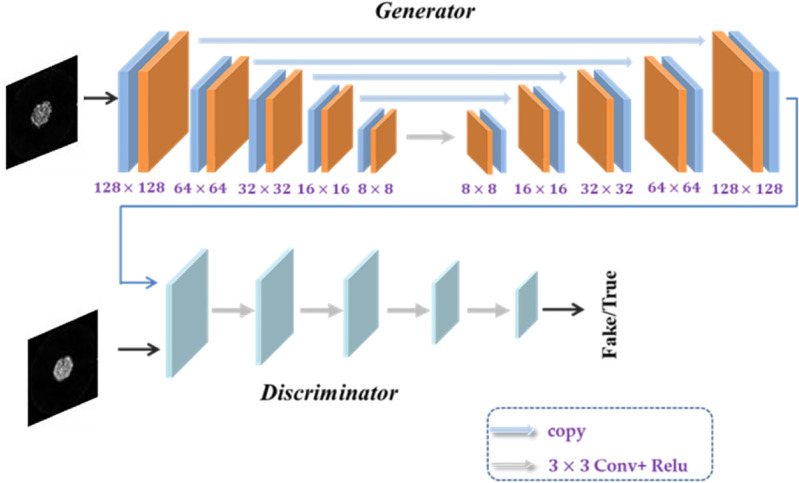
The schematic diagram of the generative adversarial networks.

We set the input to the generator is the 128×128 low-count image sample and the input to the discriminator is the same resolution high-count image. The whole net is summarized in Fig 1. The generator consists of 4 up-sampling layers and 4 down-sampling layers, and the discriminator consists of 4 down-sampling layers.

The generative nets in our method are based on the U-net structure [32] and includes batch normalization layer. The attention model is also added in the nets which inspired by the attention U-net [33]. It consists of repetitive use of (1) 3×3 convolutional layers, (2) batch normalization layers, (3) relu layers, (4) convolutional layers with stride, (5) transposed convolutional layers with stride. And in the generative nets, the major modifications are as follows: (1) fully convolutional nets are used to implement pixel segmentation, (2) attention gate is added in the left-side layer to screen features, (3) residual network is used to contact the input to the output.

**Fig 2:**
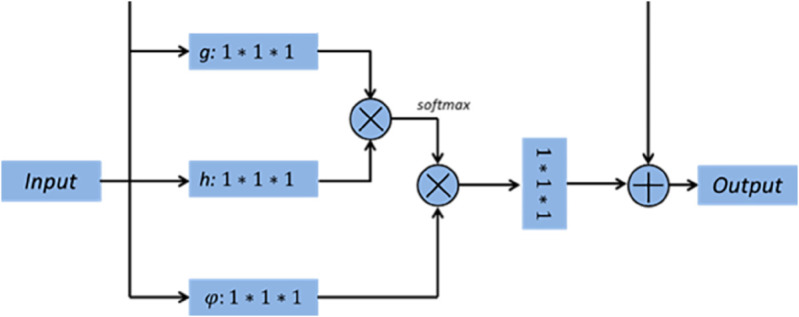
The structure of the self-attention.

The discriminator is based on the self-attention [34], and the structure is shown in Fig 2. The mechanism can directly capture the correlation features between longer distance positions in the feature map output by the upper network, which is conducive to strengthening the model’s extraction of effective image information.

**Fig 3:**
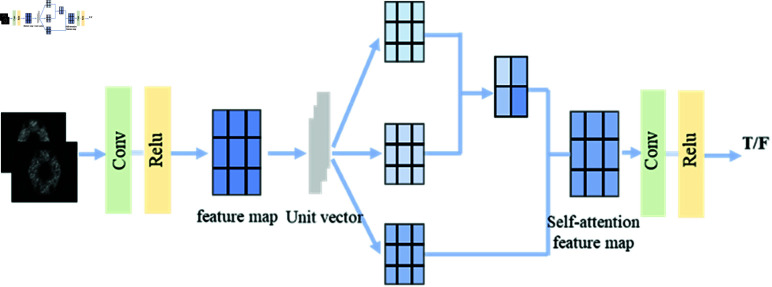
The structure of discriminative network.

The specific network structure of the discriminative network is shown in Fig 3. The self-attention layer can obtain the output weights of features at different positions of the image through training, improve the computational efficiency of the discriminant network, reduce network stacking, and accelerate model convergence. In addition, in the actual training process of the network, the embedding layers do not interfere with the dimensionality of the input data, or the network structure.

We minimize the loss function for model convergence. The loss function of the model (both the generator and the discriminator) are as Eq (9). Where *α* denotes the low-count images and x denotes the high-count images.

$$L_{G}=\eta_{1}{\left\|G(\alpha )-X\right\|}^{2}+\eta_{2}l_{bce}D(G(\alpha ))\;L_{D}=l_{bce}(D(x),1)+l_{bce}(D(x),0)\;L=L_{G}+L_{D}$$ (9)

In our implementation, we ran MLEM [35] for 25 to 35 iterations (up to general knowledge) and used its generative network output as the initial for *α* and *x*. The overall algorithm flowchart is presented in Algorithm 1. Where *m* and *n* denotes the number of max-iteration and sub-iteration, *j* means the image pixel.



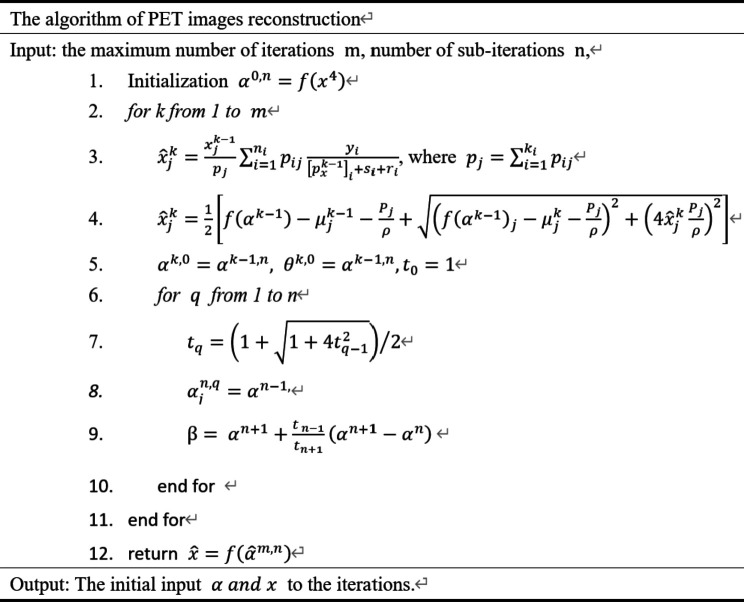



### Implement details

As the generative adversarial networks, it is inevitable that the update speed of two network parameters is inconsistent, which affects the convergence speed of the model. So, we use the TTUR (two-timescale update rule) [36] to enhance the training efficiency of the nets and that means the discriminator and the generator are given two different learning rates to balance the rate and achieve the final convergence. The learning rates are 4e-4 and 10e-4 separately for generator and discriminator by adjusting the update speed.

To avoid the over-fitting, we use dropout layers to the generator. Specifically, we trained the nets with 20% in the first three convolutional layers and the optimizer is Adam. All the networks are implemented in TensorFlow 1.0 and trained on NVIDIA GTX 1080Ti.

A. Setting of hyperparameter *λ*

The network was trained with *λ* set to 0.1,0.3,0.5,0.7 and 0.9. To reduce training time and computational costs, a randomly selected subset of 100 images was used for training and evaluation. The average sum of PSNR and SSIM was calculated to assess image quality, and the results are presented in Table 1.

**Table 1:** Performance indicators of images under different hyperparameters.

*λ*	0.1	0.3	0.5	0.7	0.9
PSNR	27.36	29.06	29.33	28.53	28.43
SSIM	0.71	0.87	0.88	0.86	0.85

According to Table 1, when *λ* is set to 0.5, the reconstructed image achieves the highest performance in both PSNR and SSIM metrics. However, as *λ* increases beyond this point, the quality of the reconstructed positron images begins to decline. Therefore, the hyperparameter *λ* in Eq (6) is ultimately set to 0.5.

B. Selection of training frequency

**Fig 4:**
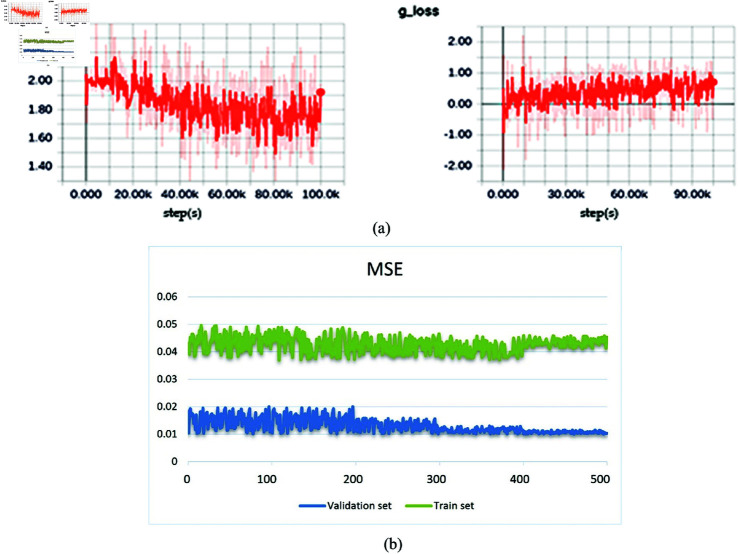
(a) The loss function in training process; (b) The training and validation mean square error.

In the process of training, we can see in Fig 4(a) that the generative model tends to converge when trained about 300 epochs (each epoch includes 1000 steps). The training and validation mean squared errors (MSE) of the reconstruction network are shown in Fig 4(b), and we can see that the validation MSE almost to minimum at about 300 epochs and the iterative reconstruction model converges.

## Experiment

### Experimental data

In this study, the experimental data are obtained from GATE simulations. GATE is a Monte Carlo-based simulation software specifically designed for PET/SPECT applications and is one of the most widely used tools in the field of nuclear medicine imaging. Built on GEANT4 (General High Energy Physics Simulation Toolkit), GATE provides well-established high-energy physics models and comprehensive geometric modeling tools, enabling accurate replication of real PET imaging conditions.

**Fig 5:**
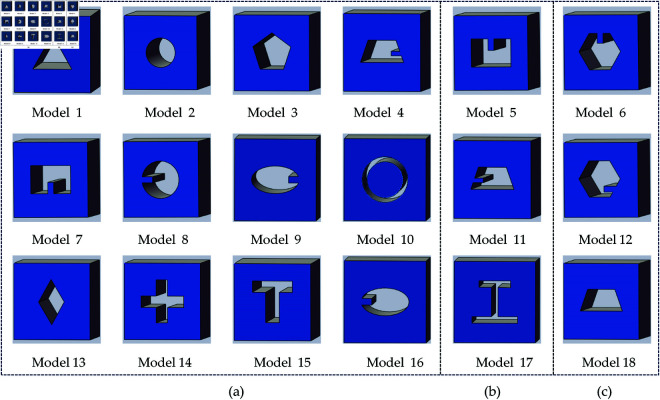
The eighteen defect template samples: (a) twelve training template samples; (b) three testing template samples; (c) three validation samples.

In our experiment, we set the data of 10 seconds scan with 800 Bq dose. For industrial cavity defects that need to be detected, we set different templates, and partial examples are shown in Fig 5. Twelve of the templates for training the model as training samples, three templates as testing samples, and three templates as verification samples. Considering the characteristics of standardization of industrial parts, the templates here are set to more regular shapes. We also use the 30 seconds scan as the high-count data. At the same time, to enhance the data of the samples, we re-sample the high count and each file contains 1/4 of the number of the list-mode.

The system matrix is calculated by linear weighting [37] and the sampling data is reconstructed by MLEM algorithm. Here, the number of iterations depends on the prior knowledge of many experiments. Within the range of iterations, we can get the reconstructed positron image which is basically consistent with the actual description.

### Experimental indicator

Here, to better describe the experimental results, we choose SSIM (structural similarity index) [38] and PSNR (Peak Signal to Noise Ratio) as the quantitative index to measure the quality of images. The indicators can be described as Eqs (10) and (11).

$$SSIM=\frac{\left(2\mu_{f}\mu_{f^{\prime }}+c_{1}\right)\left(2\sigma_{ff^{\prime }}+c_{2}\right)}{\left(\mu_{f}^{2}+\mu_{f^{\prime }}^{2}+c_{1}\right)\left(\sigma_{f}^{2}+\sigma_{f^{\prime }}^{2}+c_{2}\right)}$$ (10)

SSIM measures the similarity between two images and we can get a more intuitive comparison with structure, contrast and brightness. Where *f*(*m*, *n*) and $f^{\prime }(m,n)$ represent the pixels of reconstructed images and real images respectively; $\mu_{f}$ and $\mu_{f^{\prime }}$ are the mean value of *f*(*m*, *n*) and $f^{\prime }(m,n)$; $\sigma_{f}^{2}$ and $\sigma_{f^{\prime }}^{2}$ are the variance of *f*(*m*, *n*) and $f^{\prime }(m,n)$; $\sigma_{ff^{\prime }}$ is the covariance; *c*_1_ and *c*_2_ are the constant.

$$MSE=\frac{1}{mn}\sum_{i=0}^{m-1}\sum_{j=0}^{n-1}{\left\|I(i,j)-K(i,j)\right\|}^{2}\;PSNR=10\cdot \log_{10}\left(\frac{MAX_{I}^{2}}{MSE}\right)$$ (11)

Where *MAX*_*I*_ represents the maximum value of image color, and here the value is 255. *mn* is the size of the images and here the size is 128×128.

### Experimental results

We compared the proposed method with some other methods, and the image reconstruction are based on MLEM algorithm. The specific description of the comparative model is as follows. Here, we use two indicators, PSNR and SSIM, to quantitatively evaluate the image reconstruction results of the model, and the numerical results obtained are shown in Table 2.

- MLEM: The current mainstream traditional algorithm for positron image reconstruction;
- MLEM+CNN: Combining the most commonly used convolutional neural network in deep learning field image processing with MLEM algorithm, using CNN to train prior knowledge of positron images;
- MLEM+GAN: Combining the original GAN with the MLEM algorithm, without improving the generation network and discriminant network, especially using the original adversarial loss function as a comparative model for ablation experiments.
- MLEM+SAGAN [39]: Combining the SAGAN model with the MLEM algorithm, unlike our method, SAGAN also introduces self-attention in the generator, this situation may make the network more conducive to capturing global features, but cannot achieve a balance between geometric and texture features, which is not conducive to the characterization of positron image features.

**Table 2:** Comparison of PSNR and SSIM between the proposed method and baseline methods.

	PSNR	SSIM
MLEM	34.696	0.825
MLEM + CNN	36.367	0.832
MLEM + GAN	37.394	0.837
MLEM + SAGAN	37.389	0.840
MLEM + PIIR-GAN	38.412	0.885

It can be seen from the data in Table 2 that the method proposed in this paper improves the two basic indexes of the image to a certain extent. Moreover, by analyzing the results of experiment, it is not difficult to find that in the process of simulation data, the more complex the image is, the more sampling information it contains, and the greater the quality improvement in the reconstructed image.

**Fig 6:**
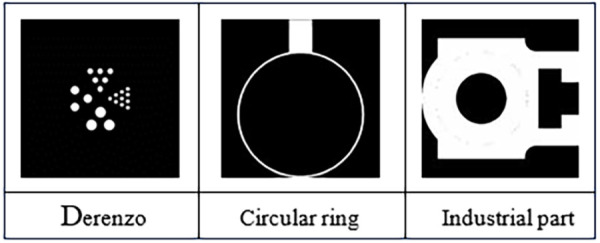
Experimental template.

In addition, further analysis of the experimental results reveals that the more complex the mechanism for detecting industrial parts, the more feature information it contains. Here, three different types of templates were designed for comparison, as shown in Fig 6.

Among them, Derenzo phantom, which includes four different cylindrical shapes with different intervals and diameters (16mm, 13mm, 8mm, and 5mm). The circular ring template is a simple circular component with protrusions, where the outer and inner radii are 80mm and 75mm respectively, and the inner protrusion is 20mm long and 10mm wide, and the last template is a general complex industrial.

**Fig 7:**
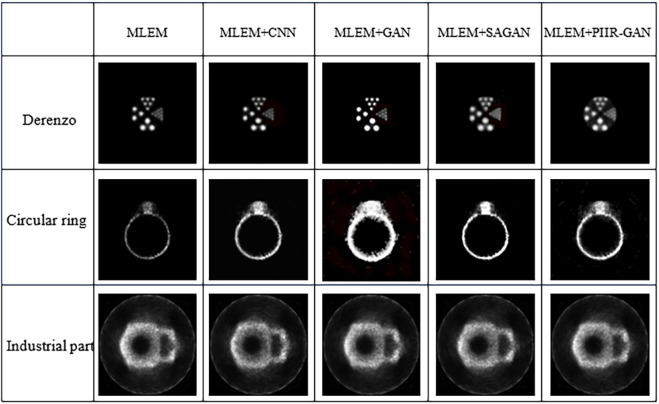
Positron reconstruction images.

The reconstructed images obtained are shown in Fig 7, and the corresponding PSNR and SSIM results are shown in Table 3. Obviously, the algorithm proposed in this article improves the quality of more complex positron reconstruction images more significantly. The PIIR-GAN algorithm introduces a self-attention mechanism module in adversarial networks, which has a better capture effect on the global features of images. However, complex positron images clearly require higher global features, thus achieving a better improvement in reconstruction quality. Furthermore, as shown in Fig 7, the MLEM lacks deep feature extraction, resulting in limited reconstruction quality. MLEM+CNN improves local detail reconstruction, but suffers from insufficient global consistency. MLEM+GAN enhances visual realism but introduces artifacts. MLEM+SAGAN captures global features, but lacks fine structure. In contrast, the proposed PIIR-GAN algorithm obtains clearer reconstruction details with almost no blurring phenomenon, and has better high-frequency structural information.

**Table 3:** Comparison of PSNR and SSIM values for reconstructed images.

	Derenzo		Circular ring		Industrial part	
	PSNR	SSIM	PSNR	SSIM	PSNR	SSIM
MLEM	28.683	0.823	28.932	0.847	30.282	0.836
MLEM + CNN	29.326	0.837	31.387	0.894	27.898	0.864
MLEM + GAN	29.928	0.856	29.491	0.876	30.127	0.897
MLEM + SAGAN	30.837	0.894	30.577	0.92	29.568	0.903
MLEM + PIIR-GAN	31.435	0.902	32.793	0.937	33.58	0.941

**Fig 8:**
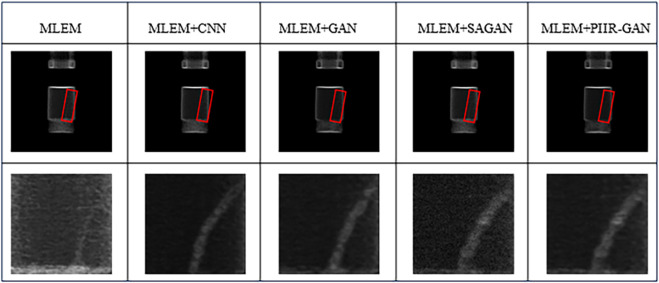
The related parameters of the hydraulic cylinder: material is alloy steel;hydraulic oil is a water glycol flame-retardant hydraulic fluid HOUGHTO-SAFE 620C; outer diameter is 55 mm; inside diameter is 45 mm; wall thickness is 5 mm.

To verify the training results and the practical reconstruction effect of our method in the field of industrial nondestructive testing, we design a group of experiments based on the industrial parts of hydraulic cylinder. Hydraulic cylinder can convert hydraulic energy into mechanical energy, which is widely used in various mechanical hydraulic systems. Therefore, it has good application value to carry out the actual test. In the experiment, the PET detector we used was Trans-PET Explorist 180, with a detector crystal resolution of 1 millimeter. The hydraulic component is made of aluminum, with an outer diameter of 55mm, an inner diameter of 45mm, a wall thickness of 5mm, and an axial length of 20cm. We cut a groove with a depth of 3mm at the pipe wall of the hydraulic component to simulate possible cracks. During the experiment, approximately 350 milliliters of radioactive mixture with a configuration of 1.85 mCi were injected. The reconstruction results under different comparative models are shown in Fig 8.

From the local enlarged drawing, we can see the crack shape of the inner wall of the hydraulic parts. Combined with the specific size of the hydraulic parts (Length, diameter, etc.), we can further determine the location and size of the crack. Industrial parts generally have standard size parameters, so the operability of this method is extremely high. Here, it also needs to be explained, due to the limitations of current hardware conditions, the resolution of the image needs to be improved. Of course, in the later researches, we can also strengthen the research on the post-processing stage of positron reconstruction image, especially in the aspect of edge processing, so as to detect the position of defects more accurately in the post-processing stage of image, and better achieve the purpose of industrial nondestructive testing.

## Discussion

At present, our application of PET technology in the field of industrial non-destructive testing is mainly focused on the gaps in complex cavities and the description of the internal flow field of industrial parts. Many existing works have used pure MLEM algorithm, simple convolutional neural network or other ways to obtain better images. Here, we use Generative Adversarial Networks as the sampling data representation and the model is added in the iterative reconstruction stage. Thus, compared with other methods, especially in the post-processing stage, the proposed method is constrained in the data sampling which can retain finer information features to avoid the loss of details in the reconstruction process. At the same time, in the iterative reconstruction process, it can be found that as the number of iterations increases, the image quality will not be significantly improved, and may even lead to an increase in noise. Therefore, we ensure the stability of the model through regular optimization of the network.

In addition, we observed that the adversarial learning mechanism in PIIR-GAN effectively improves the model’s ability to distinguish signal from noise, leading to a clearer reconstruction of structural boundaries. This advantage becomes more pronounced under low-sampling conditions, where traditional iterative or CNN-based methods tend to produce blurred edges or over-smoothed textures. The integration of prior information through the generator ensures that the reconstructed images preserve both global consistency and local details, which is essential for accurately identifying defect morphology in industrial components.

**Fig 9:**
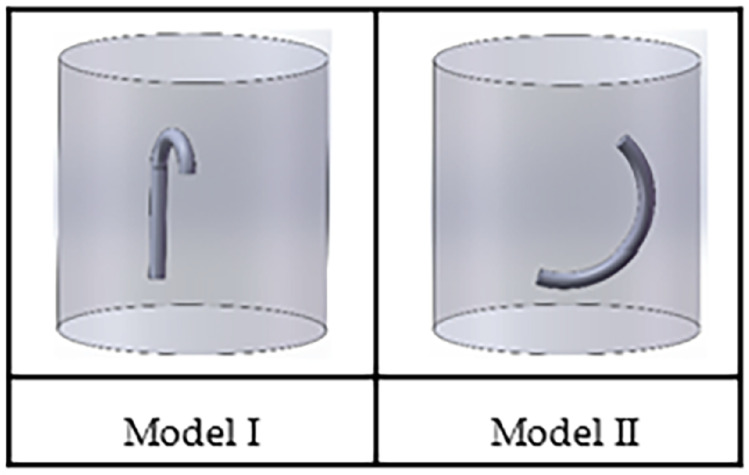
SolidWorks simulation model.

In addition, we designed a more targeted experiment to prove the superiority of the proposed method in image detail feature processing. Two different sets of models were designed using SolidWorks in the experiment to simulate the possible presence of foreign objects of different shapes in the inner cavity of a circular pipeline. The models are shown as Fig 9.

**Fig 10:**
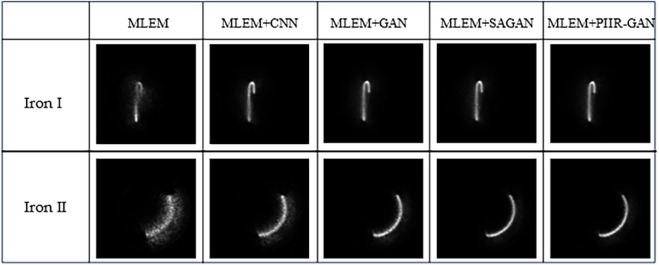
Experimental parameters: the concentration of nuclide is 800 bq; the sampling time is 10 s; the material is the iron wire (foreign body) in the cavity.

And the comparison model in the experiment is the same as above, and the final positron image obtained is shown in Fig 10.

The above two groups of positron images are obtained in the practical application. Specifically, the same shape of the cavity is detected. From the experimental results, we can see that our method can describe the wire more clearly and completely, and there is no or very little image layer breaking phenomenon in the middle, and the experimental effect is not affected by the different shapes of the wire. And the quantitative comparison is shown in Table 4. Clearly, PIIR-GAN has shown significant improvements in PSNR and SSIM metrics and have also shown good performance improvements in practical industrial detecting.Specifically, PIIR-GAN achieved an average PSNR improvement of 3.3dB and a 6% increase in SSIM compared with the baseline GAN model, confirming its enhanced image fidelity and structural preservation. Overall, these results validate that incorporating adversarial learning into the iterative PET reconstruction framework provides both quantitative and qualitative benefits, making it more suitable for industrial non-destructive testing scenarios.

**Table 4:** Comparison of PSNR and SSIM values for reconstructed images.

	Iron 1		Iron 2	
	PSNR	SSIM	PSNR	SSIM
MLEM	29.479	0.834	27.418	0.908
MLEM + CNN	33.552	0.923	31.552	0.929
MLEM + GAN	33.436	0.927	32.538	0.937
MLEM + SAGAN	34.821	0.94	34.941	0.969
MLEM + PIIR-GAN	36.733	0.987	37.328	0.982

## Conclusions

Considering the objective difficulties faced by positron imaging technology in industrial non-destructive testing applications, such as short adoption time and limited data, to solve the problem of poor image reconstruction quality, this paper proposes an industrial positron image iterative reconstruction algorithm that integrates generative adversarial networks. This algorithm can significantly improve the quality of positron image reconstruction and has strong domain applicability. PIIR-GAN showed that combined with the deep neural network, adding prior knowledge to MLEM reconstructing algorithm can better adapt to the specific conditions on industrial non-destructive testing. In particular, we introduced a self-attention mechanism in the network to make PET image reconstruction in line with actual image characteristics (A large number of experiments show that the same set of PET detection device, that is, under the same physical parameters, the network structure is basically fixed and not affected by the images).

However, the proposed approach still has some limitations that would need to be addressed. First, considering the short sampling time and the difficulty in data collection, the network model needs to be further improved. Second, most experiments are under simulation conditions, and more field experiments are needed to optimize the entire reconstruction algorithm network. In addition, on the basis of optimizing the image reconstruction algorithm, we also need to consider the use of denoising, edge detection and other methods in the subsequent processing stage of the image to improve the final presentation quality of the image, so as to achieve more accurate detection. In the future, more work will focus on improving the quality of reconstructed images. We plan to study more advanced neural network models and further perfect the images dataset. Moreover, the three-dimensional reconstruction of images is also the focus of the subsequent research.

## Supporting information

S1 Data We have uploaded the data set as a Supporting information file named “data.zip”. (ZIP)
