# Supplementary figures and images for: Iterative reconstruction of industrial positron images with generative networks (part 2 of 2)
Source: PLoS One. 2025 Nov 19;20(11):e0335912. doi: 10.1371/journal.pone.0335912 (PMC12629474; doi:10.1371/journal.pone.0335912)

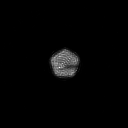

Supplement: S1 Data — (ZIP) [file pone.0335912.s001.zip › data/0105.png]

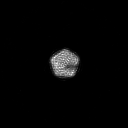

Supplement: S1 Data — (ZIP) [file pone.0335912.s001.zip › data/0106.png]

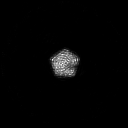

Supplement: S1 Data — (ZIP) [file pone.0335912.s001.zip › data/0107.png]

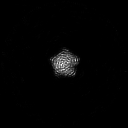

Supplement: S1 Data — (ZIP) [file pone.0335912.s001.zip › data/0108.png]

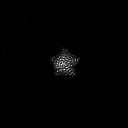

Supplement: S1 Data — (ZIP) [file pone.0335912.s001.zip › data/0109.png]

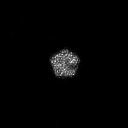

Supplement: S1 Data — (ZIP) [file pone.0335912.s001.zip › data/0110.png]

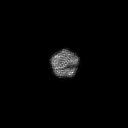

Supplement: S1 Data — (ZIP) [file pone.0335912.s001.zip › data/0111.png]

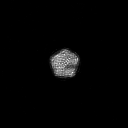

Supplement: S1 Data — (ZIP) [file pone.0335912.s001.zip › data/0112.png]

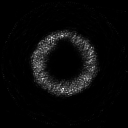

Supplement: S1 Data — (ZIP) [file pone.0335912.s001.zip › data/0113.png]

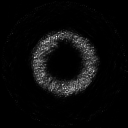

Supplement: S1 Data — (ZIP) [file pone.0335912.s001.zip › data/0114.png]

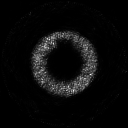

Supplement: S1 Data — (ZIP) [file pone.0335912.s001.zip › data/0115.png]

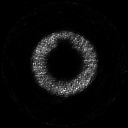

Supplement: S1 Data — (ZIP) [file pone.0335912.s001.zip › data/0116.png]

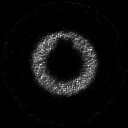

Supplement: S1 Data — (ZIP) [file pone.0335912.s001.zip › data/0117.png]

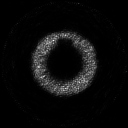

Supplement: S1 Data — (ZIP) [file pone.0335912.s001.zip › data/0118.png]

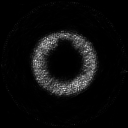

Supplement: S1 Data — (ZIP) [file pone.0335912.s001.zip › data/0119.png]

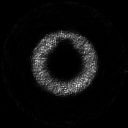

Supplement: S1 Data — (ZIP) [file pone.0335912.s001.zip › data/0120.png]

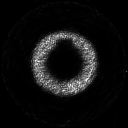

Supplement: S1 Data — (ZIP) [file pone.0335912.s001.zip › data/0121.png]

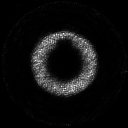

Supplement: S1 Data — (ZIP) [file pone.0335912.s001.zip › data/0122.png]

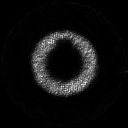

Supplement: S1 Data — (ZIP) [file pone.0335912.s001.zip › data/0123.png]

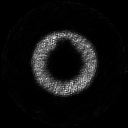

Supplement: S1 Data — (ZIP) [file pone.0335912.s001.zip › data/0124.png]

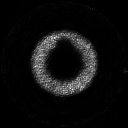

Supplement: S1 Data — (ZIP) [file pone.0335912.s001.zip › data/0125.png]

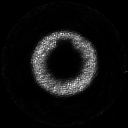

Supplement: S1 Data — (ZIP) [file pone.0335912.s001.zip › data/0126.png]

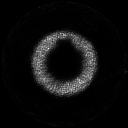

Supplement: S1 Data — (ZIP) [file pone.0335912.s001.zip › data/0127.png]

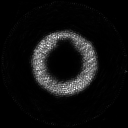

Supplement: S1 Data — (ZIP) [file pone.0335912.s001.zip › data/0128.png]

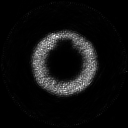

Supplement: S1 Data — (ZIP) [file pone.0335912.s001.zip › data/0129.png]

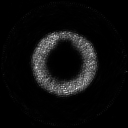

Supplement: S1 Data — (ZIP) [file pone.0335912.s001.zip › data/0130.png]

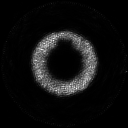

Supplement: S1 Data — (ZIP) [file pone.0335912.s001.zip › data/0131.png]

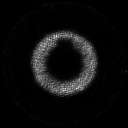

Supplement: S1 Data — (ZIP) [file pone.0335912.s001.zip › data/0132.png]

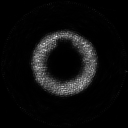

Supplement: S1 Data — (ZIP) [file pone.0335912.s001.zip › data/0133.png]

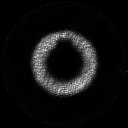

Supplement: S1 Data — (ZIP) [file pone.0335912.s001.zip › data/0134.png]

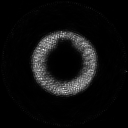

Supplement: S1 Data — (ZIP) [file pone.0335912.s001.zip › data/0135.png]

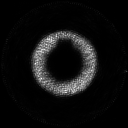

Supplement: S1 Data — (ZIP) [file pone.0335912.s001.zip › data/0136.png]

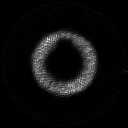

Supplement: S1 Data — (ZIP) [file pone.0335912.s001.zip › data/0137.png]

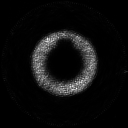

Supplement: S1 Data — (ZIP) [file pone.0335912.s001.zip › data/0138.png]

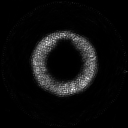

Supplement: S1 Data — (ZIP) [file pone.0335912.s001.zip › data/0139.png]

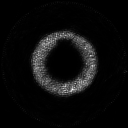

Supplement: S1 Data — (ZIP) [file pone.0335912.s001.zip › data/0140.png]

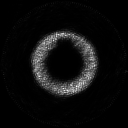

Supplement: S1 Data — (ZIP) [file pone.0335912.s001.zip › data/0141.png]

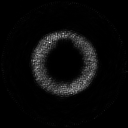

Supplement: S1 Data — (ZIP) [file pone.0335912.s001.zip › data/0142.png]

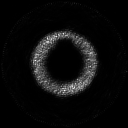

Supplement: S1 Data — (ZIP) [file pone.0335912.s001.zip › data/0143.png]

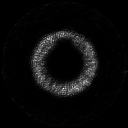

Supplement: S1 Data — (ZIP) [file pone.0335912.s001.zip › data/0144.png]

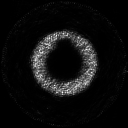

Supplement: S1 Data — (ZIP) [file pone.0335912.s001.zip › data/0145.png]

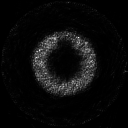

Supplement: S1 Data — (ZIP) [file pone.0335912.s001.zip › data/0146.png]

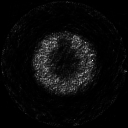

Supplement: S1 Data — (ZIP) [file pone.0335912.s001.zip › data/0147.png]

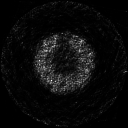

Supplement: S1 Data — (ZIP) [file pone.0335912.s001.zip › data/0148.png]

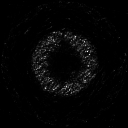

Supplement: S1 Data — (ZIP) [file pone.0335912.s001.zip › data/0149.png]

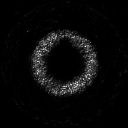

Supplement: S1 Data — (ZIP) [file pone.0335912.s001.zip › data/0150.png]

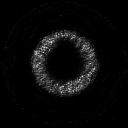

Supplement: S1 Data — (ZIP) [file pone.0335912.s001.zip › data/0151.png]

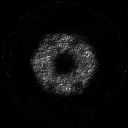

Supplement: S1 Data — (ZIP) [file pone.0335912.s001.zip › data/0152.png]

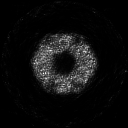

Supplement: S1 Data — (ZIP) [file pone.0335912.s001.zip › data/0153.png]

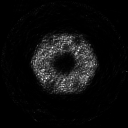

Supplement: S1 Data — (ZIP) [file pone.0335912.s001.zip › data/0154.png]

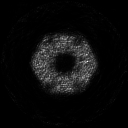

Supplement: S1 Data — (ZIP) [file pone.0335912.s001.zip › data/0155.png]

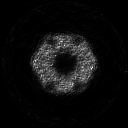

Supplement: S1 Data — (ZIP) [file pone.0335912.s001.zip › data/0156.png]

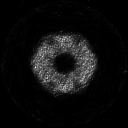

Supplement: S1 Data — (ZIP) [file pone.0335912.s001.zip › data/0157.png]

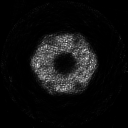

Supplement: S1 Data — (ZIP) [file pone.0335912.s001.zip › data/0158.png]

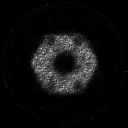

Supplement: S1 Data — (ZIP) [file pone.0335912.s001.zip › data/0159.png]

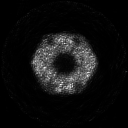

Supplement: S1 Data — (ZIP) [file pone.0335912.s001.zip › data/0160.png]

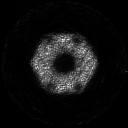

Supplement: S1 Data — (ZIP) [file pone.0335912.s001.zip › data/0161.png]

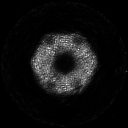

Supplement: S1 Data — (ZIP) [file pone.0335912.s001.zip › data/0162.png]

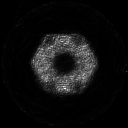

Supplement: S1 Data — (ZIP) [file pone.0335912.s001.zip › data/0163.png]

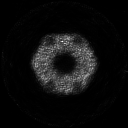

Supplement: S1 Data — (ZIP) [file pone.0335912.s001.zip › data/0164.png]

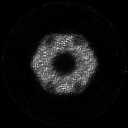

Supplement: S1 Data — (ZIP) [file pone.0335912.s001.zip › data/0165.png]

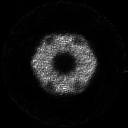

Supplement: S1 Data — (ZIP) [file pone.0335912.s001.zip › data/0166.png]

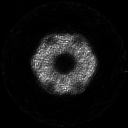

Supplement: S1 Data — (ZIP) [file pone.0335912.s001.zip › data/0167.png]

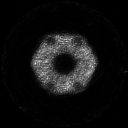

Supplement: S1 Data — (ZIP) [file pone.0335912.s001.zip › data/0168.png]

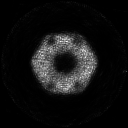

Supplement: S1 Data — (ZIP) [file pone.0335912.s001.zip › data/0169.png]

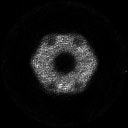

Supplement: S1 Data — (ZIP) [file pone.0335912.s001.zip › data/0170.png]

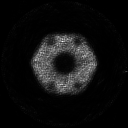

Supplement: S1 Data — (ZIP) [file pone.0335912.s001.zip › data/0171.png]

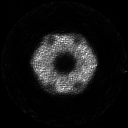

Supplement: S1 Data — (ZIP) [file pone.0335912.s001.zip › data/0172.png]

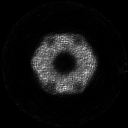

Supplement: S1 Data — (ZIP) [file pone.0335912.s001.zip › data/0173.png]

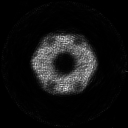

Supplement: S1 Data — (ZIP) [file pone.0335912.s001.zip › data/0174.png]

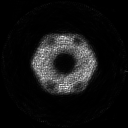

Supplement: S1 Data — (ZIP) [file pone.0335912.s001.zip › data/0175.png]

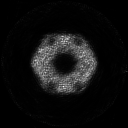

Supplement: S1 Data — (ZIP) [file pone.0335912.s001.zip › data/0176.png]

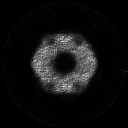

Supplement: S1 Data — (ZIP) [file pone.0335912.s001.zip › data/0177.png]

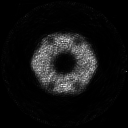

Supplement: S1 Data — (ZIP) [file pone.0335912.s001.zip › data/0178.png]

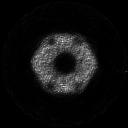

Supplement: S1 Data — (ZIP) [file pone.0335912.s001.zip › data/0179.png]

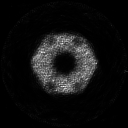

Supplement: S1 Data — (ZIP) [file pone.0335912.s001.zip › data/0180.png]

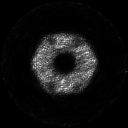

Supplement: S1 Data — (ZIP) [file pone.0335912.s001.zip › data/0181.png]

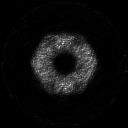

Supplement: S1 Data — (ZIP) [file pone.0335912.s001.zip › data/0182.png]

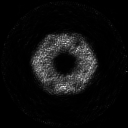

Supplement: S1 Data — (ZIP) [file pone.0335912.s001.zip › data/0183.png]

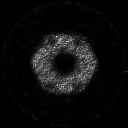

Supplement: S1 Data — (ZIP) [file pone.0335912.s001.zip › data/0184.png]

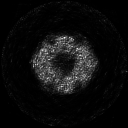

Supplement: S1 Data — (ZIP) [file pone.0335912.s001.zip › data/0185.png]

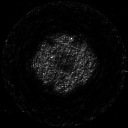

Supplement: S1 Data — (ZIP) [file pone.0335912.s001.zip › data/0186.png]

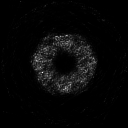

Supplement: S1 Data — (ZIP) [file pone.0335912.s001.zip › data/0187.png]

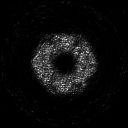

Supplement: S1 Data — (ZIP) [file pone.0335912.s001.zip › data/0188.png]

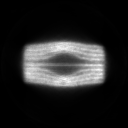

Supplement: S1 Data — (ZIP) [file pone.0335912.s001.zip › data/0189.png]

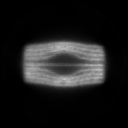

Supplement: S1 Data — (ZIP) [file pone.0335912.s001.zip › data/0190.png]

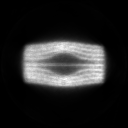

Supplement: S1 Data — (ZIP) [file pone.0335912.s001.zip › data/0191.png]

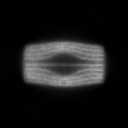

Supplement: S1 Data — (ZIP) [file pone.0335912.s001.zip › data/0192.png]

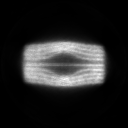

Supplement: S1 Data — (ZIP) [file pone.0335912.s001.zip › data/0193.png]

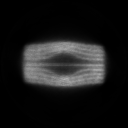

Supplement: S1 Data — (ZIP) [file pone.0335912.s001.zip › data/0194.png]

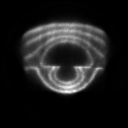

Supplement: S1 Data — (ZIP) [file pone.0335912.s001.zip › data/0195.png]

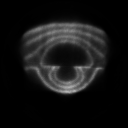

Supplement: S1 Data — (ZIP) [file pone.0335912.s001.zip › data/0196.png]

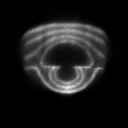

Supplement: S1 Data — (ZIP) [file pone.0335912.s001.zip › data/0197.png]

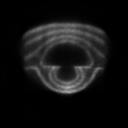

Supplement: S1 Data — (ZIP) [file pone.0335912.s001.zip › data/0198.png]

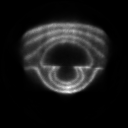

Supplement: S1 Data — (ZIP) [file pone.0335912.s001.zip › data/0199.png]

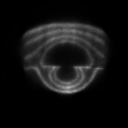

Supplement: S1 Data — (ZIP) [file pone.0335912.s001.zip › data/0200.png]
