# Supplementary figures and images for: Iterative reconstruction of industrial positron images with generative networks (part 1 of 2)
Source: PLoS One. 2025 Nov 19;20(11):e0335912. doi: 10.1371/journal.pone.0335912 (PMC12629474; doi:10.1371/journal.pone.0335912)

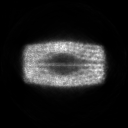

Supplement: S1 Data — (ZIP) [file pone.0335912.s001.zip › data/0001.png]

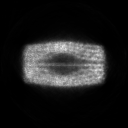

Supplement: S1 Data — (ZIP) [file pone.0335912.s001.zip › data/0002.png]

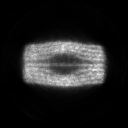

Supplement: S1 Data — (ZIP) [file pone.0335912.s001.zip › data/0003.png]

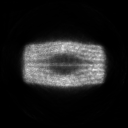

Supplement: S1 Data — (ZIP) [file pone.0335912.s001.zip › data/0004.png]

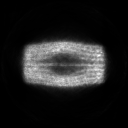

Supplement: S1 Data — (ZIP) [file pone.0335912.s001.zip › data/0005.png]

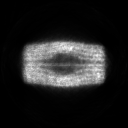

Supplement: S1 Data — (ZIP) [file pone.0335912.s001.zip › data/0007.png]

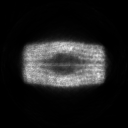

Supplement: S1 Data — (ZIP) [file pone.0335912.s001.zip › data/0008.png]

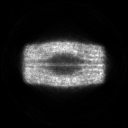

Supplement: S1 Data — (ZIP) [file pone.0335912.s001.zip › data/0009.png]

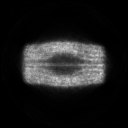

Supplement: S1 Data — (ZIP) [file pone.0335912.s001.zip › data/0010.png]

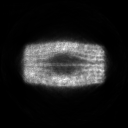

Supplement: S1 Data — (ZIP) [file pone.0335912.s001.zip › data/0011.png]

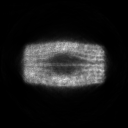

Supplement: S1 Data — (ZIP) [file pone.0335912.s001.zip › data/0012.png]

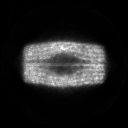

Supplement: S1 Data — (ZIP) [file pone.0335912.s001.zip › data/0013.png]

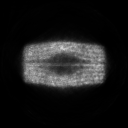

Supplement: S1 Data — (ZIP) [file pone.0335912.s001.zip › data/0014.png]

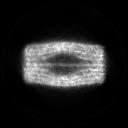

Supplement: S1 Data — (ZIP) [file pone.0335912.s001.zip › data/0015.png]

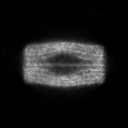

Supplement: S1 Data — (ZIP) [file pone.0335912.s001.zip › data/0016.png]

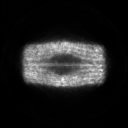

Supplement: S1 Data — (ZIP) [file pone.0335912.s001.zip › data/0017.png]

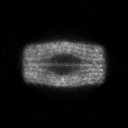

Supplement: S1 Data — (ZIP) [file pone.0335912.s001.zip › data/0018.png]

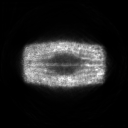

Supplement: S1 Data — (ZIP) [file pone.0335912.s001.zip › data/0019.png]

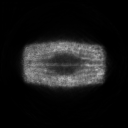

Supplement: S1 Data — (ZIP) [file pone.0335912.s001.zip › data/0020.png]

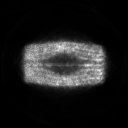

Supplement: S1 Data — (ZIP) [file pone.0335912.s001.zip › data/0021.png]

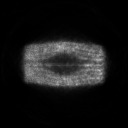

Supplement: S1 Data — (ZIP) [file pone.0335912.s001.zip › data/0022.png]

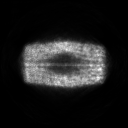

Supplement: S1 Data — (ZIP) [file pone.0335912.s001.zip › data/0023.png]

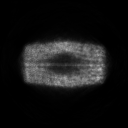

Supplement: S1 Data — (ZIP) [file pone.0335912.s001.zip › data/0024.png]

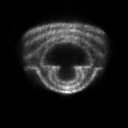

Supplement: S1 Data — (ZIP) [file pone.0335912.s001.zip › data/0025.png]

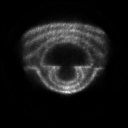

Supplement: S1 Data — (ZIP) [file pone.0335912.s001.zip › data/0026.png]

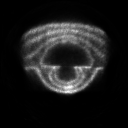

Supplement: S1 Data — (ZIP) [file pone.0335912.s001.zip › data/0027.png]

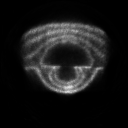

Supplement: S1 Data — (ZIP) [file pone.0335912.s001.zip › data/0028.png]

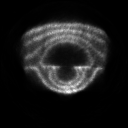

Supplement: S1 Data — (ZIP) [file pone.0335912.s001.zip › data/0029.png]

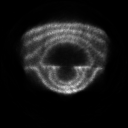

Supplement: S1 Data — (ZIP) [file pone.0335912.s001.zip › data/0030.png]

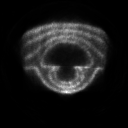

Supplement: S1 Data — (ZIP) [file pone.0335912.s001.zip › data/0031.png]

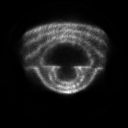

Supplement: S1 Data — (ZIP) [file pone.0335912.s001.zip › data/0033.png]

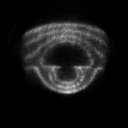

Supplement: S1 Data — (ZIP) [file pone.0335912.s001.zip › data/0034.png]

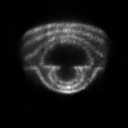

Supplement: S1 Data — (ZIP) [file pone.0335912.s001.zip › data/0035.png]

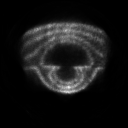

Supplement: S1 Data — (ZIP) [file pone.0335912.s001.zip › data/0036.png]

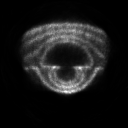

Supplement: S1 Data — (ZIP) [file pone.0335912.s001.zip › data/0037.png]

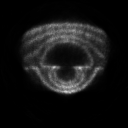

Supplement: S1 Data — (ZIP) [file pone.0335912.s001.zip › data/0038.png]

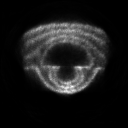

Supplement: S1 Data — (ZIP) [file pone.0335912.s001.zip › data/0039.png]

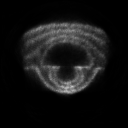

Supplement: S1 Data — (ZIP) [file pone.0335912.s001.zip › data/0040.png]

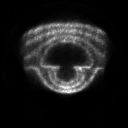

Supplement: S1 Data — (ZIP) [file pone.0335912.s001.zip › data/0041.png]

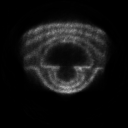

Supplement: S1 Data — (ZIP) [file pone.0335912.s001.zip › data/0042.png]

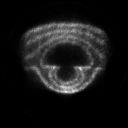

Supplement: S1 Data — (ZIP) [file pone.0335912.s001.zip › data/0043.png]

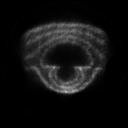

Supplement: S1 Data — (ZIP) [file pone.0335912.s001.zip › data/0044.png]

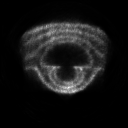

Supplement: S1 Data — (ZIP) [file pone.0335912.s001.zip › data/0045.png]

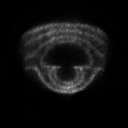

Supplement: S1 Data — (ZIP) [file pone.0335912.s001.zip › data/0046.png]

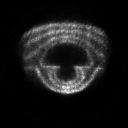

Supplement: S1 Data — (ZIP) [file pone.0335912.s001.zip › data/0047.png]

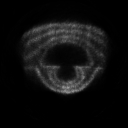

Supplement: S1 Data — (ZIP) [file pone.0335912.s001.zip › data/0048.png]

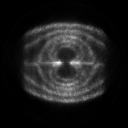

Supplement: S1 Data — (ZIP) [file pone.0335912.s001.zip › data/0049.png]

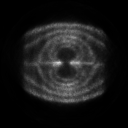

Supplement: S1 Data — (ZIP) [file pone.0335912.s001.zip › data/0050.png]

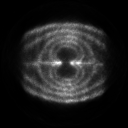

Supplement: S1 Data — (ZIP) [file pone.0335912.s001.zip › data/0051.png]

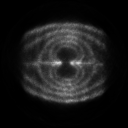

Supplement: S1 Data — (ZIP) [file pone.0335912.s001.zip › data/0052.png]

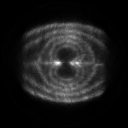

Supplement: S1 Data — (ZIP) [file pone.0335912.s001.zip › data/0053.png]

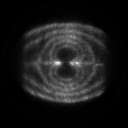

Supplement: S1 Data — (ZIP) [file pone.0335912.s001.zip › data/0054.png]

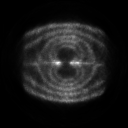

Supplement: S1 Data — (ZIP) [file pone.0335912.s001.zip › data/0055.png]

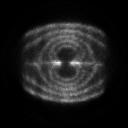

Supplement: S1 Data — (ZIP) [file pone.0335912.s001.zip › data/0057.png]

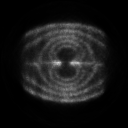

Supplement: S1 Data — (ZIP) [file pone.0335912.s001.zip › data/0058.png]

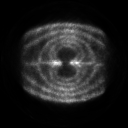

Supplement: S1 Data — (ZIP) [file pone.0335912.s001.zip › data/0059.png]

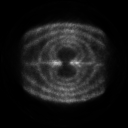

Supplement: S1 Data — (ZIP) [file pone.0335912.s001.zip › data/0060.png]

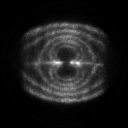

Supplement: S1 Data — (ZIP) [file pone.0335912.s001.zip › data/0061.png]

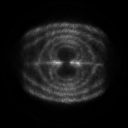

Supplement: S1 Data — (ZIP) [file pone.0335912.s001.zip › data/0062.png]

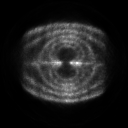

Supplement: S1 Data — (ZIP) [file pone.0335912.s001.zip › data/0063.png]

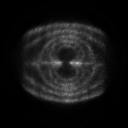

Supplement: S1 Data — (ZIP) [file pone.0335912.s001.zip › data/0064.png]

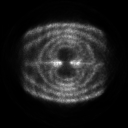

Supplement: S1 Data — (ZIP) [file pone.0335912.s001.zip › data/0065.png]

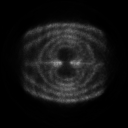

Supplement: S1 Data — (ZIP) [file pone.0335912.s001.zip › data/0066.png]

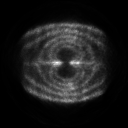

Supplement: S1 Data — (ZIP) [file pone.0335912.s001.zip › data/0067.png]

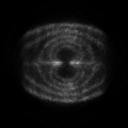

Supplement: S1 Data — (ZIP) [file pone.0335912.s001.zip › data/0068.png]

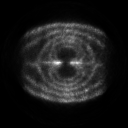

Supplement: S1 Data — (ZIP) [file pone.0335912.s001.zip › data/0069.png]

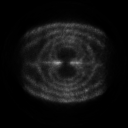

Supplement: S1 Data — (ZIP) [file pone.0335912.s001.zip › data/0070.png]

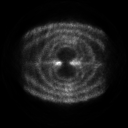

Supplement: S1 Data — (ZIP) [file pone.0335912.s001.zip › data/0071.png]

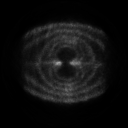

Supplement: S1 Data — (ZIP) [file pone.0335912.s001.zip › data/0072.png]

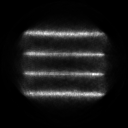

Supplement: S1 Data — (ZIP) [file pone.0335912.s001.zip › data/0073.png]

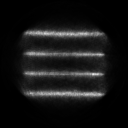

Supplement: S1 Data — (ZIP) [file pone.0335912.s001.zip › data/0074.png]

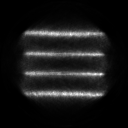

Supplement: S1 Data — (ZIP) [file pone.0335912.s001.zip › data/0075.png]

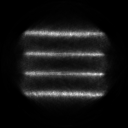

Supplement: S1 Data — (ZIP) [file pone.0335912.s001.zip › data/0076.png]

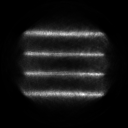

Supplement: S1 Data — (ZIP) [file pone.0335912.s001.zip › data/0077.png]

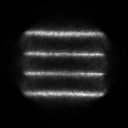

Supplement: S1 Data — (ZIP) [file pone.0335912.s001.zip › data/0079.png]

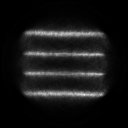

Supplement: S1 Data — (ZIP) [file pone.0335912.s001.zip › data/0080.png]

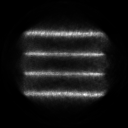

Supplement: S1 Data — (ZIP) [file pone.0335912.s001.zip › data/0081.png]

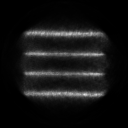

Supplement: S1 Data — (ZIP) [file pone.0335912.s001.zip › data/0082.png]

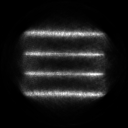

Supplement: S1 Data — (ZIP) [file pone.0335912.s001.zip › data/0083.png]

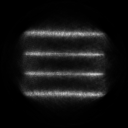

Supplement: S1 Data — (ZIP) [file pone.0335912.s001.zip › data/0084.png]

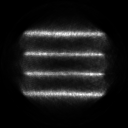

Supplement: S1 Data — (ZIP) [file pone.0335912.s001.zip › data/0085.png]

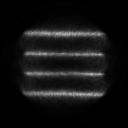

Supplement: S1 Data — (ZIP) [file pone.0335912.s001.zip › data/0086.png]

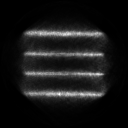

Supplement: S1 Data — (ZIP) [file pone.0335912.s001.zip › data/0087.png]

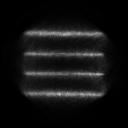

Supplement: S1 Data — (ZIP) [file pone.0335912.s001.zip › data/0088.png]

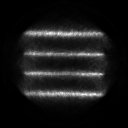

Supplement: S1 Data — (ZIP) [file pone.0335912.s001.zip › data/0089.png]

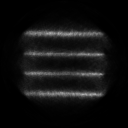

Supplement: S1 Data — (ZIP) [file pone.0335912.s001.zip › data/0090.png]

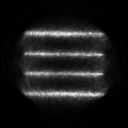

Supplement: S1 Data — (ZIP) [file pone.0335912.s001.zip › data/0091.png]

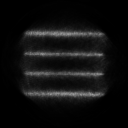

Supplement: S1 Data — (ZIP) [file pone.0335912.s001.zip › data/0092.png]

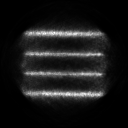

Supplement: S1 Data — (ZIP) [file pone.0335912.s001.zip › data/0093.png]

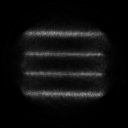

Supplement: S1 Data — (ZIP) [file pone.0335912.s001.zip › data/0094.png]

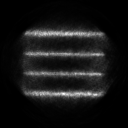

Supplement: S1 Data — (ZIP) [file pone.0335912.s001.zip › data/0095.png]

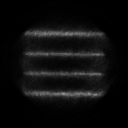

Supplement: S1 Data — (ZIP) [file pone.0335912.s001.zip › data/0096.png]

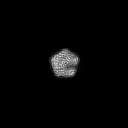

Supplement: S1 Data — (ZIP) [file pone.0335912.s001.zip › data/0097.png]

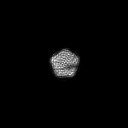

Supplement: S1 Data — (ZIP) [file pone.0335912.s001.zip › data/0098.png]

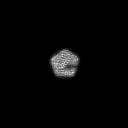

Supplement: S1 Data — (ZIP) [file pone.0335912.s001.zip › data/0099.png]

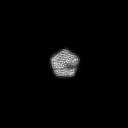

Supplement: S1 Data — (ZIP) [file pone.0335912.s001.zip › data/0100.png]

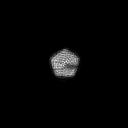

Supplement: S1 Data — (ZIP) [file pone.0335912.s001.zip › data/0101.png]

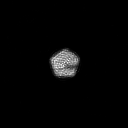

Supplement: S1 Data — (ZIP) [file pone.0335912.s001.zip › data/0102.png]

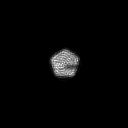

Supplement: S1 Data — (ZIP) [file pone.0335912.s001.zip › data/0103.png]

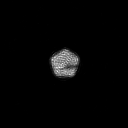

Supplement: S1 Data — (ZIP) [file pone.0335912.s001.zip › data/0104.png]
